# Segmental and global longitudinal strain measurement by 2-dimensional speckle tracking echocardiography in severe rheumatic mitral stenosis

**DOI:** 10.1186/s12872-023-03624-x

**Published:** 2023-11-27

**Authors:** Samira Mehrabi-Pari, Sepehr Nayebirad, Akbar Shafiee, Ahmad Vakili-Basir, Reza Hali, Mojgan Ghavami, Arash Jalali

**Affiliations:** 1 Tehran Heart Center, Cardiovascular Diseases Research Institute, Tehran University of Medical Sciences, Tehran, Iran; 2 Department of Epidemiology and Biostatistics, School of Public Health, Tehran University of Medical Sciences, Tehran, Iran; 3 Department of Echocardiography, Tehran Heart Center, North Karegar st, Tehran, 1411713138 Iran

**Keywords:** Rheumatic Heart Disease, Mitral stenosis, Left ventricular dysfunction, Strain measurement

## Abstract

**Background:**

The present study aimed to detect subtle left ventricular (LV) dysfunction in patients with severe rheumatic mitral stenosis (MS) by measuring global and segmental longitudinal strain with a two-dimensional speckle tracking echocardiography (2D-STE) method.

**Methods:**

In this case-control study, 65 patients with severe rheumatic MS and preserved ejection fraction (EF ≥ 50% measured by conventional echocardiographic methods) were compared with 31 otherwise healthy control subjects. All patients underwent LV strain measurement by the 2D-STE method in addition to conventional echocardiography using a VIVID S60 echocardiography device.

**Results:**

Absolute strain values in myocardial segments 1–8, 10, and 12 (all basal, mid anterior, mid anteroseptal, mid inferior, and mid anterolateral segments) were significantly lower in patients with severe MS compared with the control group (P < 0.05 for all). The absolute global longitudinal strain (GLS) value was higher in the control group (-19.56 vs. -18.25; P = 0.006). After adjustment for age, gender, and systolic blood pressure, the difference in GLS between the two groups was as follows: mean difference=-1.16; 95% CI: -2.58–0.25; P = 0.110.

**Conclusion:**

In patients with severe rheumatic MS and preserved EF, the absolute GLS tended to be lower than healthy controls. Furthermore, the segmental strain values of LV were significantly lower in most of the basal and some mid-myocardial segments. Further studies are warranted to investigate the underlying pathophysiology and clinical implications of this subclinical dysfunction in certain segments of patients with severe rheumatic MS.

## Introduction

Mitral stenosis (MS) is a hemodynamic abnormality of the mitral valve, usually resulting from thickened valve leaflets with restricted motion impeding normal blood flow [1]. In developing countries, MS is commonly caused by rheumatic heart disease, an autoimmune reaction to untreated streptococcal infection [2]. Previous studies have demonstrated that patients with MS may have concomitant left ventricular (LV) systolic dysfunction in conventional echocardiography using two-dimensional (2D) and M-mode measurements [3, 4].

Evaluation of LV ejection fraction (LVEF) is a simple global measurement of ventricular function. Still, it is associated with many limitations, such as dependency on the operator performing the study, the patient’s volume loading condition, and the chamber’s geometry. In addition, in many patients, using only routine parameters of conventional echocardiography is insufficient to demonstrate subtle LV dysfunction of the disease [5].

Speckle tracking echocardiography (STE) is a method of assessing cardiac function in which an echocardiographic device with the capability of identifying and tracking a certain point in the myocardium with specific echocardiographic characteristics within a block of speckles is used to determine the displacement of this speckle through the cardiac cycle. STE can calculate parameters like longitudinal, radial, or circumferential myocardial strain, global longitudinal strain (GLS), and strain rate [6–9]. However, longitudinal strain is generally believed to be a better predictor for various outcomes than other myocardial strain indices [10, 11]. Despite this advantage, segmental longitudinal strain measurement may be subject to high variability and intervendor bias compared to GLS. Nevertheless, it can be utilized alongside GLS to provide a more complete picture of myocardial strain [12]. GLS measurement is a sensitive method to evaluate LV dysfunction that can demonstrate myocardial abnormality without any reduction in ejection fraction (EF), and it can be used in risk stratification of patients [13–15].

Therefore, in the current study, we aimed to detect the presence and distribution of subclinical LV dysfunction in patients with severe MS but preserved EF by segmental and global longitudinal strain measurement using 2D-STE method.

## Methods

### Study population

The target population of our study was patients with severe rheumatic MS who were referred to the echocardiography laboratory at our medical center from June 2020 to June 2021. The inclusion criteria were EF ≥ 50% and echocardiographic features of rheumatic mitral valve stenosis. Exclusion criteria included atrial fibrillation rhythm, known coronary artery disease, concomitant moderate or severe mitral regurgitation, aortic stenosis or insufficiency (according to 2020 ACC/AHA guidelines) [16], previous balloon mitral valvotomy (BMV) or any open cardiac surgery, end-stage renal failure, hepatic cirrhosis, metastatic cancer, diabetes or uncontrolled hypertension, poor standard echocardiographic views to obtain all needed variables as well as beta-blocker therapy. In addition, patients with cardiomyopathies such as hypertrophic cardiomyopathy or cardiac amyloidosis, and bundle branch or any other conduction block in ECG were not included.

A total of 65 patients with severe rheumatic MS were selected as the case group, and 31 otherwise healthy subjects as the control group. The study protocol was approved by the Ethics Committee of Tehran University of Medical Sciences (IR.TUMS.THC.REC.1399.035) and was performed under the Declaration of Helsinki. All patients provided written informed consent before enrollment.

#### Examinations

Demographic characteristics were registered for all subjects. Systolic and diastolic blood pressure (SBP and DBP) were measured from both arms after 15 min of rest in a sitting position, and the mean values were recorded. Heart rate was counted in one minute after 15 min of rest.

Complete trans-thoracic echocardiography, including strain study by 2D speckle tracking method in two, three, and four-chamber views, was performed by an experienced echocardiologist using the same echocardiography device (VIVID S60, GE Healthcare, US with 3SC transducer) for all participants. We used the modified Simpson method (biplane method of disks) with the tracing of LV cavity area in both apical four-chamber and apical two-chamber views to measure LVEF according to ASE recommendations [17]. The analysis was performed on the scanner directly using automated function imaging. The software version was Echopac 202.

Echocardiographic data, including other valvular heart diseases, data related to mitral annular tissue velocities (septal é and lateral é), LV end-systolic and end-diastolic dimensions and left atrial area (by biplane area-length method) were all obtained for both groups. In addition, the mitral valve area (MVA) was measured by 2D planimetry, and the Wilkins score was calculated for the case group. MS severity was determined based on the MVA measurement. Patients with MVA < 1.5 cm² were included in the study (severe MS) [18].

**Fig. 1:**
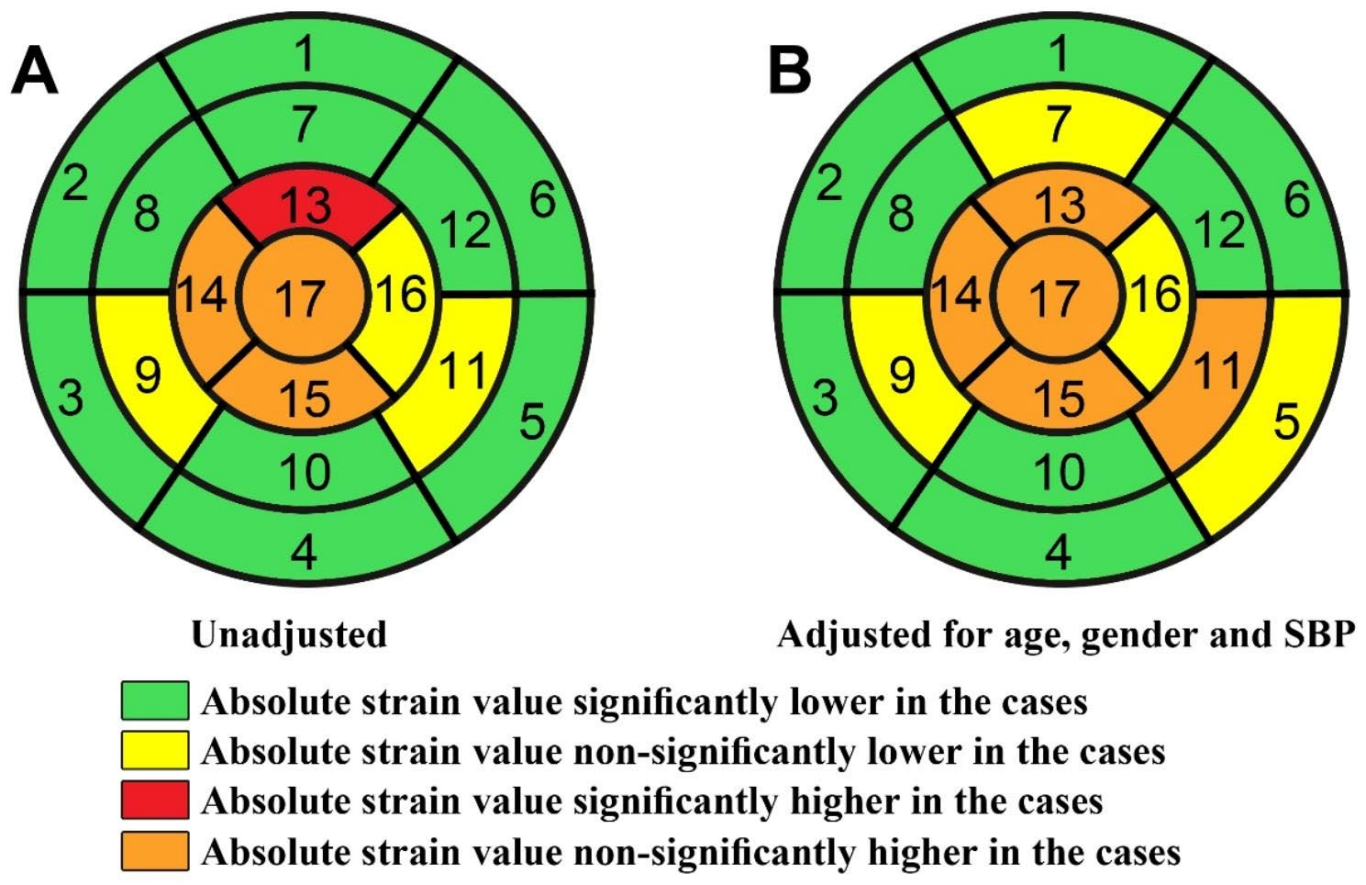
Unadjusted (**A**) and adjusted (**B**) effect of severe MS on segmental longitudinal strains. In the green and yellow segments, the absolute strain value was lower in the rheumatic MS group than in the controls

**Fig. 2:**
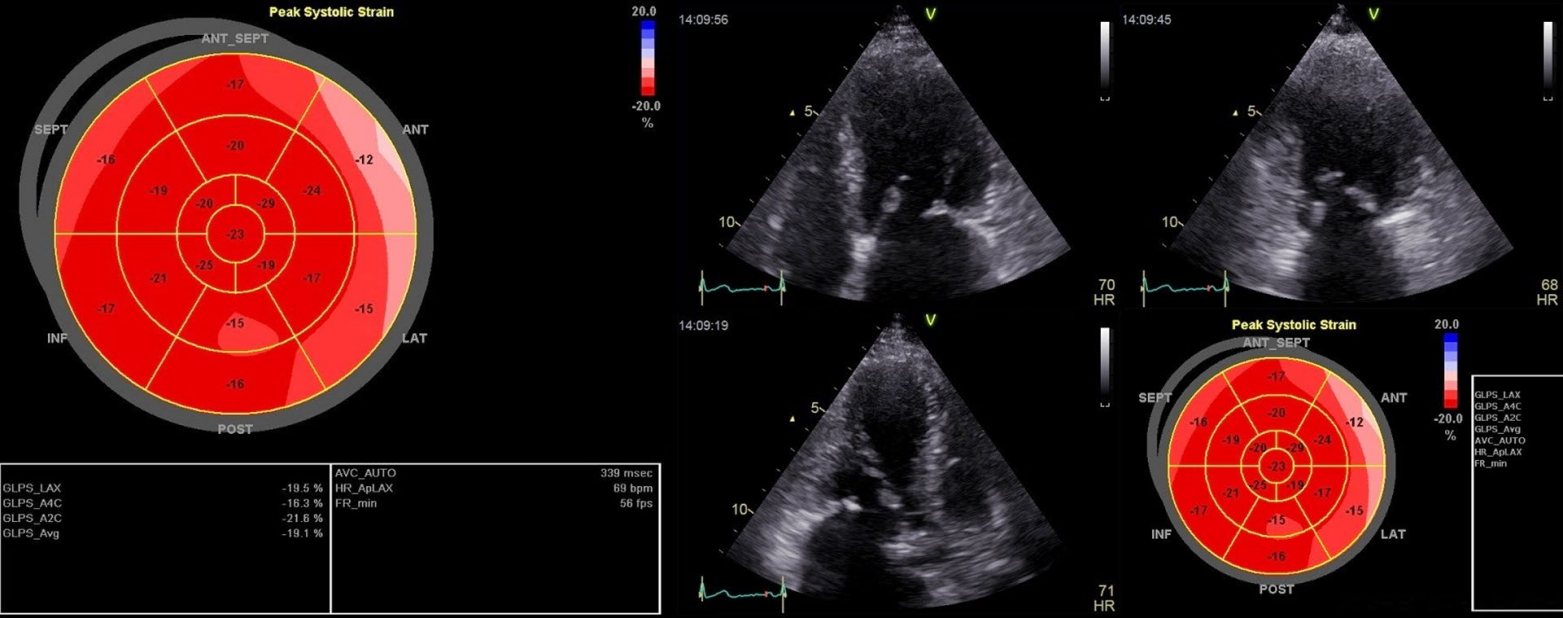
Bull’s eye plot in a patient with severe rheumatic MS showing reduced segmental longitudinal strain values in basal and some mid myocardial segments despite good global longitudinal strain. As presented in the image, three 2-D echocardiographic views, including 4, 3, and 2 chamber apical views with a focus on LV, were used to assess global and longitudinal strain values. To perform, in each view, three points were plotted manually at the base of each opposing wall as well as the apex using a point-and-click technique, and then the endocardial border was automatically traced by the software. The software divides the region of interest in each view into six segments, and the quality of tracing of each segment is automatically scored as either acceptable or unacceptable with the possibility of further correction manually. Finally, the software calculates global and segmental strains, displaying the values. (The figure is only for demonstration of the technique and was not taken from an included patient in the study)

Segmental longitudinal strain measurement was performed in both groups using the 2D-STE method. GLS was calculated by automatically averaging the strain values of all myocardial segments obtained by the device [7, 19]. Segmentation of LV myocardium was based on the American Heart Association’s (AHA) 17-segment model as follows: (1) Basal anterior (2) Basal anteroseptal (3) Basal inferoseptal (4) Basal inferior (5) Basal inferolateral (6) Basal anterolateral (7) Mid anterior (8) Mid anteroseptal (9) Mid inferoseptal (10) Mid inferior 11. Mid inferolateral 12. Mid anterolateral 13. Apical anterior 14. Apical septal 15. Apical inferior 16. Apical lateral 17. Apex (Fig. 1) [20]. Figure 2 demonstrates our strain measurement method using Bull’s eye plot in a patient with severe rheumatic MS. The image was taken after the study completion and is from a patient referred to our center but not included in this study.

### Statistical analysis

Continuous variables were presented as mean (standard deviation) and were compared between two groups using the independent t-test. Categorical variables were expressed as frequency (percentage) and were compared between groups applying Chi^2^ or Fisher’s exact test, as appropriate. The adjusted effect of severe MS on segmental and global longitudinal strain was evaluated using an ANOVA-ANCOVA model. Age, gender, and SBP were considered potential confounders, so the adjustment was applied for these items.

Cohen’d and Partial Eta squared were also reported. Since all patients with severe MS in this study had moderate Wilkins scores between 8 and 12, no adjustment was applied based on this score. The sample size was measured using an alpha error probability of 5%, statistical power of 95%, and “effect size d” of 0.75. A 2:1 ratio (cases: controls) was employed for sample size calculation, resulting in 59 cases and 29 controls. Sample size calculation was performed using G power software version 3.1. All other statistical analyses were conducted with IBM SPSS for Windows, version 23.0 (IBM Corp., Armonk, N.Y., USA).

## Results

From 210 MS patients referred to our echocardiography lab, 65 (30.9%) were included. We analyzed 65 severe MS patients (83% female, 54 ± 11 years old) and 31 control subjects (48% female, 42 ± 12). Severe MS patients had an MVA of 1.15 ± 0.21 cm^2^ by 2D planimetry, a mean gradient of 7.12 ± 3.24 mmHg by Doppler assessment, and a Wilkins score of 9.48 ± 0.92. The average heart rate was 70.49 ± 14.20 at the time of gradient assessment. Aortic insufficiency (AI) and mitral regurgitation (MR) with less than moderate severity were more prevalent in patients with severe MS in comparison with the control group (*p* value < 0.001). Subjects with moderate or more severe mitral or aortic regurgitation were already excluded from the study. Tissue velocities were significantly lower in severe MS patients than in normal subjects (P < 0.001). Additionally, the left atrial area was significantly larger in severe MS patients compared to the controls (25.48 cm² vs. 15.13 cm², P < 0.001, Table 1**).**

**Table 1:** Demographic, clinical, and echocardiographic characteristics of cases and controls

	Total	Control (N = 31)	Rheumatic MS (N = 65)	*p* value
Female	69 (71.9%)	15 (48.4%)	54 (83.1%)	**< 0.001**
Age (year)	50.35 (12.39)	42.38 (11.68)	54.15 (10.89)	**< 0.001**
SBP (mmHg)	125.17 (20.41)	120.51 (16.49)	127.38 (21.80)	0.124
DBP (mmHg)	74.96 (10.54)	71.35 (9.78)	76.67 (10.52)	**0.020**
h (beat per minute)	70.72 (13.65)	71.19 (12.62)	70.49 (14.20)	0.815
Ejection fraction	54.56 (1.99)	55.08 (1.77)	54.31 (2.05)	0.075
***AI***				**< 0.001**
No	24 (25%)	14 (45.2%)	10 (15.4%)	
Trivial	28 (29.2%)	13 (41.9%)	15 (23.1%)	
Mild	28 (29.2%)	4 (12.9%)	24 (36.9%)	
Mild to moderate	16 (16.7%)	0 (0%)	16 (24.6%)	
***MR***				**< 0.001**
No	14 (14.6%)	14 (45.2%)	0 (0%)	
Trivial	17 (17.7%)	11 (35.5%)	6 (9.2%)	
Mild	47 (49%)	6 (19.4%)	41 (63.1%)	
Mild to moderate	18 (18.8%)	0 (0%)	18 (27.7%)	
***AS***				**0.171**
No	91 (94.8%)	31 (100%)	60 (92.3%)	
Mild	5 (5.2%)	0 (0%)	5 (7.7%)	
és (cm/s)	9.38 (2.21)	10.94 (1.26)	8.65 (2.18)	**< 0.001**
élat (cm/s)	9.26 (3.49)	13.42 (1.93)	7.28 (1.99)	**< 0.001**
LVEDD (mm)	48.94 (4.08)	48.94 (4.13)	48.97 (4.09)	0.913
LVESD (mm)	34.10 (4.78)	33.84 (4.80)	34.23 (4.80)	0.709
LAA (cm^2^)	22.14 (6.29)	15.13 (2.11)	25.48 (4.65)	**< 0.001**

AI = aortic insufficiency; AS = aortic stenosis; SBP = systolic blood pressure; DBP = diastolic blood pressure; élat= é lateral; és= é septal; HR = heart rate; LAA = left atrial area; LVEDD = left ventricle end-diastolic dimension; LVESD = left ventricle end-systolic dimension; MR = Mitral regurgitation; MS = mitral stenosis; continuous variables are reported as means (standard deviation) and categorical variables as frequency (percentage%)

Absolute value for GLS and strain in myocardial segments 1–8, 10, and 12 (all basal, mid anterior, mid anteroseptal, mid inferior, and mid anterolateral segments) were significantly lower in patients with severe MS in comparison with the controls (P < 0.05 for all), while only in segment 13 (apical anterior segment) segmental strain in the case group showed higher absolute value than the control group. Differences in other segments were not statistically significant **(**Table 2**).** Figure 1 compares the differences in strain values of different heart segments between the case and control groups.

**Table 2:** Segmental and global longitudinal strain measurements in the case and control groups

	Total	Control (N = 31)	Rheumatic MS (N = 65)	Mean Difference	LLCI	ULCI	*p* value	Cohen’s d
Seg1, Basal anterior	-14.94 (4.58)	-17.16 (2.49)	-13.88 (4.97)	-3.28	-4.80	-1.77	**< 0.001**	-0.76
Seg2, Basal anteroseptal	-13.20 (4.13)	-16.90 (2.41)	-11.43 (3.58)	-5.47	-6.71	-4.24	**< 0.001**	-1.68
Seg3, Basal inferoseptal	-16.20 (3.25)	-17.90 (2.06)	-15.38 (3.40)	-2.52	-3.63	-1.40	**< 0.001**	-0.83
Seg4, Basal inferior	-15.73 (4.40)	-17.59 (3.13)	-14.84 (4.66)	-2.73	-4.34	-1.13	**0.001**	-0.65
Seg5, Basal inferolateral	-16.33 (4.02)	-17.65 (2.97)	-15.70 (4.32)	-1.94	-3.44	-0.43	**0.012**	-0.49
Seg6, Basal anterolateral	-15.10 (4.53)	-18.22 (3.24)	-13.62 (4.31)	-4.61	-6.18	-3.04	**< 0.001**	-1.15
Seg7, Mid anterior	-17.30 (5.15)	-18.49 (2.77)	-16.72 (5.89)	-1.76	-3.52	-0.01	**0.049**	-0.34
Seg8, Mid anteroseptal	-17.80 (3.16)	-19.45 (2.39)	-17.02 (3.19)	-2.44	-3.6	-1.27	**< 0.001**	-0.82
Seg9, Mid inferoseptal	-19.46 (3.18)	-19.94 (2.34)	-19.23 (3.50)	-0.70	-1.91	0.50	0.247	-0.22
Seg10, Mid inferior	-17.86 (4.30)	-19.74 (2.76)	-16.95 (4.62)	-2.79	-4.29	-1.28	**< 0.001**	-0.68
Seg11, Mid inferolateral	-18.80 (4.33)	-19.45 (2.87)	-18.49 (4.87)	-0.96	-2.54	0.62	0.230	-0.22
Seg12, Mid anterolateral	-17.78 (4.37)	-20.65 (2.76)	-16.42 (4.35)	-4.23	-5.69	-2.77	**< 0.001**	-1.08
Seg13, Apical anterior	-22.44 (4.62)	-20.90 (2.94)	-23.19 (5.10)	2.29	0.63	3.94	**0.007**	2.28
Seg14, Apical septal	-22.61 (4.58)	-21.94 (2.98)	-22.94 (5.17)	1.00	-0.66	2.67	0.235	0.22
Seg15, Apical inferior	-22.15 (5.01)	-21.97 (3.43)	-22.23 (5.64)	0.27	-1.59	2.13	0.777	0.05
Seg16, Apical lateral	-21.72 (4.96)	-22.55 (2.81)	-21.32 (5.70)	-1.23	-2.98	0.51	0.165	-0.25
Seg17, Apex	-22.26 (4.22)	-22.03 (2.99)	-22.37 (4.71)	0.34	-1.24	1.91	0.672	0.08
GLS	-18.67 (2.66)	-19.56 (1.60)	-18.25 (2.96)	-1.31	-2.24	-0.39	**0.006**	-0.50

GLS = global longitudinal strain; seg = segment; Data are reported as means (standard deviation); LLCI = lower limit of 95% confidence interval; ULCI = upper limit of 95% confidence interval; MS = mitral stenosis

On average, the absolute value of GLS in severe MS patients was 1.31 lower than in controls (P = 0.006, 95%CI: -2.24 – -0.39, Cohen’s d= -0.5**)**. The differences in segments 1–8, 10, and 12 were also statistically significant. Cohen’s d analysis also showed medium to large effect size differences in these segments. After statistical adjustment, the absolute value of GLS in severe MS patients was still 1.16 lower than in control subjects, but this difference was not statistically significant anymore (P = 0.110, 95% CI: -2.58–0.25). Differences in segments 1, 2, 3, 4, 6, 8, 10, and 12 were still significant after adjustment for confounding factors **(**Tables 2 and 3**)**.

**Table 3:** Mean difference of segmental and global longitudinal strains after adjusting for possible confounders

Variables	Adjusted				
	Mean Difference	LLCI	ULCI	*p* value	Partial eta squared
Seg1, Basal anterior	-3.41	-5.74	-1.08	**0.008**	0.134
Seg2, Basal anteroseptal	-5.28	-7.14	-3.41	**< 0.001**	0.290
Seg3, Basal inferoseptal	-1.80	-3.42	-0.18	**0.030**	0.510
Seg4, Basal inferior	-2.50	-4.80	-0.21	**0.033**	0.490
Seg5, Basal inferolateral	-1.27	-3.39	0.85	0.240	0.150
Seg6, Basal anterolateral	-4.60	-6.75	-2.45	**< 0.001**	0.170
Seg7, Mid anterior	-1.81	-4.54	0.92	0.192	0.019
Seg8, Mid anteroseptal	-2.88	-4.48	-1.29	**< 0.001**	0.124
Seg9, Mid inferoseptal	-1.10	-2.82	0.60	0.200	0.018
Seg10, Mid inferior	-2.45	-4.68	-0.22	**0.032**	0.050
Seg11, Mid inferolateral	0.14	-2.18	2.45	0.910	0.001
Seg12, Mid anterolateral	-3.56	-5.66	-1.47	**0.001**	0.110
Seg13, Apical anterior	2.65	-0.17	4.74	0.068	0.040
Seg14, Apical septal	1.27	-1.22	3.76	0.320	0.011
Seg15, Apical inferior	1.40	-1.30	4.10	0.310	0.120
Seg16, Apical lateral	-0.87	-3.54	1.80	0.510	0.005
Seg17, Apex	0.54	-1.74	2.82	0.640	0.002
GLS	-1.16	-2.58	0.25	0.110	0.030

GLS = global longitudinal strain; LLCI = lower limit of 95% confidence interval; ULCI = upper limit of 95% confidence interval; Seg = segment; Data are reported as means (standard deviation). Mean difference = mean value of the controls – mean value of the cases. The adjustment was performed for age, gender, and systolic blood pressure using an ANOVA-ANCOVA model

## Discussion

In brief, we conducted a case-control study of MS patients and otherwise healthy subjects, all with normal EF. Patients underwent 2D-STE exam. Longitudinal strain and GLS were measured and compared between the two groups. The absolute average value for GLS was lower in MS patients than in controls but did not reach statistical significance. On the other hand, the absolute strain values for basal and mid-myocardial segments were significantly lower in the MS subjects.

It is estimated that MS has a prevalence of 51.21 per 100,000 in developing countries [21]. It has been suggested that a subset of MS patients, despite normal EF, may have some degree of LV dysfunction [22]. This myocardial dysfunction can be detected in STE by a lower absolute value of segmental and global strain [13–15]. Gerede et al. proposed that MS patients with a GLS value of under − 16.98 or a GLS rate of under − 1.45 had a faster disease progression [23]. This highlights the need for closer follow-up in MS patients with lower strain rates.

In a study by Sengupta et al., 57 patients with severe MS and 19 healthy controls underwent 2D speckle tracking-based GLS measurement. Patients with severe MS had lower LVEF and absolute GLS values than the control group. GLS in 48 (84.2%) patients with severe MS was below the 25th percentile of controls. After BMV, GLS was significantly improved (before: -14.6 ± 3.3% vs. after: -17.8 ± 3.5%) [24]. In a similar study on 72 patients with isolated MS and 31 controls, no significant difference was found in LVEF and LV end-systolic or diastolic dimensions between the two groups. Absolute GLS in patients with MS was significantly lower than in the control group. Interestingly, no significant differences in GLS measurements among MS sub-groups (mild, moderate, and severe) were reported [25].

In our study, MS patients had lower absolute strain values compared to controls in the basal and middle segments (1–4, 6, 8, 10, and 12). While some studies have had similar results, others have reported differences in strain values of all segments. For instance, Simsek et al. study on 32 patients with isolated MS and 25 healthy controls demonstrated that absolute values of peak systolic strain and strain rate of all heart segments in patients with MS were significantly lower than in healthy subjects. This study used a different method of heart segmentation with 12 segments [26]. On the other hand, Ozdemir et al. showed that patients with severe MS had a reduced longitudinal peak strain and strain rate in all basal and some mid-segments of the left ventricle [27]. In Roushdy et al. investigation, left and right ventricular GLS measurements were performed on 32 patients with MS and 30 healthy subjects. It noted that left and right ventricular absolute GLS values in patients with MS were lower than the control group (-16.5 ± 2.7% vs. -21.0 ± 1.5 and − 18.3 ± 4.7 vs. -19.8 ± 1.3, respectively). The strain measurements in basal and septal segments and RV free wall were significantly decreased. After BMV, examinations were repeated within 24 h and three months later in patients with MS. Compared with baseline values, left and right ventricular absolute GLS were significantly increased within 24 h after BMV. This increase was maintained after three months [22].

LV dysfunction is frequently observed in MS patients [28]. Although LV dysfunction can present with reduced EF, some MS patients may have subclinical LV dysfunction with preserved EF. The exact underlying mechanisms are still up for debate. Some studies, like Sengupta et al., suggested that LV dysfunction is mainly due to hemodynamic abnormality of the heart and can be reversible after valvulotomy [24]. A possible explanation is valvulotomy increases the preload, thus increasing LV end-diastolic volume (LVED). Increased LVED is then believed to improve LV function. Other studies suggest that there may be an intrinsic LV abnormality in MS that may not be completely reversible with valvulotomy [29]. McKay et al. showed that there was either no change or minimal increase in LVED volume after balloon valvuloplasty in MS patients [30]. These findings contradict the reduced LVED theory [28, 31]. Regardless of the mechanism, LV-GLS measurement can aid in detecting subclinical LV dysfunction in rheumatic MS patients with preserved EF.

There is also some pathological evidence on the topic. Waller et al. described the histology of excised stenotic mitral valves and reported evidence of Aschoff bodies in the interstitial tissue of the myocardium [32]. Although the clinical significance of Aschoff bodies is unknown, Waller et al. suggested that they may represent previous fibrinoid necrosis in the heart. This hypothesis may also be reasonable from a pathophysiological point of view. Rheumatic MS is a delayed complication of acute rheumatic fever attack involving all three heart layers (endocardium, myocardium, and pericardium). Although the attack is acute, the inflammation appears to continue and cause fibrosis and scarring of the mitral apparatus. It may be presumable that similar mechanisms also result in endocardium and myocardium fibrosis, as Klein et al. suggested [28]. It has been hypothesized that this fibrosis may extend from the mitral apparatus to the adjacent myocardium, explaining the difference in strain values of basal segments between MS patients and the normal population [22, 27, 33]. Still, there is no conclusive evidence confirming these assumptions. Another hypothesis is that the fibrotic mitral valve can cause poster-basal wall motion abnormality by having a tethering effect [34].

It is noteworthy that segments 13–15 and 17 showed slightly higher absolute strains in severe MS patients than in controls. While the difference in the strain value of these segments was mainly negligible, the strain value for segment 13 was notably higher in the cases. We believe this contradictory finding in this single myocardial segment may be due to some technical error in accurate image acquisition of the apical-anterior segment in the apical two-chamber view and should be assessed more in future studies. Another explanation is that there may be inaccuracies in tracking because of poor image quality or the operator missing control of the tracking.

### Limitations

A major limitation of the current study was the non-matched case-control design. ANOVA-ANCOVA statistical adjustment was used to overcome the important differences in baseline characteristics, and the final results were mainly expectable and compatible with the pathophysiology of rheumatic MS. In addition, we showed that while the absolute strain value was lower in the basolateral segments in the cases compared to controls, in some apical segments, such as segment 13, it might be higher among the cases. Thus, it is presumable that the GLS index alone may not be accurate enough to identify myocardial dysfunction, and longitudinal strain of other segments is required to provide a complete picture.

Another limitation was the rather small sample size of the study, which stems from the rarity of severe rheumatic MS patients. However, this was a common shortcoming among similar studies on this topic. Additionally, intraobserver variability was not measured in the current study. Finally, the imaging artifacts due to respiration may have affected strain measurements’ quality. To solve this issue, we performed image acquisitions at the end of expiration with three registered cycles.

## Conclusion

In patients with severe rheumatic MS and preserved EF, the absolute GLS tended to be lower than healthy controls. Furthermore, the segmental strain values of LV were significantly lower in most of the basal and some mid-myocardial segments. Further studies are warranted to investigate the underlying pathophysiology and clinical implications of this subclinical dysfunction in certain segments of patients with severe rheumatic MS.

## Data Availability

The datasets generated and/or analyzed during the current study are not publicly available due to Tehran Heart Center’s policy but are available from the corresponding author upon reasonable request.
